# Acute effect of traditional and adaptive metronomes on gait variability in older individuals with a history of falls

**DOI:** 10.1007/s40520-021-02066-9

**Published:** 2022-01-12

**Authors:** Anna Cronström, Michael H. Cole, Daniel Chalkley, Steven Van Andel, Gert-Jan Pepping, Mark W. Creaby

**Affiliations:** 1 School of Behavioural and Health Sciences, Australian Catholic University, Brisbane, Australia; 2 Department of Health Sciences, Lund University, Lund, Sweden; 3 Department of Community Medicine and Rehabilitation, Umeå University, Umeå, Sweden; 4 Healthy Brain & Mind Research Centre, Australian Catholic University, Melbourne, Australia; 5 Department of Sport Science, University of Innsbruck, Innsbruck, Austria

**Keywords:** Fall prevention, Gait, Variability, Biofeedback, Adaptive metronome

## Abstract

**Background:**

Metronome cueing has been shown to reduce gait variability and thereby potentially reduce falls risk in individuals with Parkinson’s disease. It is unclear however, if metronome cueing has a similar effect in healthy older adults with a history of falls.

**Aim:**

To investigate whether a traditional and/or an adaptive metronome, based on an individual’s gait pattern, were effective in reducing gait variability in older adults with a history of falls.

**Methods:**

Twenty older adults (15 women, 71 ± 4.9 years) with a history of falls were included in this cross-over study. Participants received two types of cueing (adaptive and traditional metronome) 1 week apart. The variability of the participants’ stride time, stride length, walking speed and duration of double leg support were recorded during three walking conditions (baseline, during feedback and post-feedback gait). Repeated-measures ANOVA was used to assess the possible effects of the two cueing strategies on gait variables.

**Results:**

Compared with the baseline condition, participants had significantly increased stride time variability during feedback (*F* (2) = 9.83, *p* < 0.001) and decreased double leg support time variability post-feedback (*F* (2) 3.69, *p* = 0.034). Increased stride time variability was observed with the adaptive metronome in comparison to the traditional metronome.

**Conclusion:**

Metronome cueing strategies may reduce double leg support variability in older adults with a history of falls but seem to increase stride time variability. Further studies are needed to investigate if metronome cueing is more beneficial for individuals with greater baseline gait variability than those included in the current study.

## Background

Thirty percent of community-dwelling individuals aged 65 years and older will fall at least once each year, significantly increasing their risk of injury, loss of independence, hospitalization and mortality [1–3]. Between 34 and 67% of all falls experienced by older adults occur during walking [4–8], suggesting that gait deficits may account for a large percentage of these falls. Older adults who have a history of falls walk slower, take shorter steps, spend more time in double leg support and exhibit more variable stride lengths and stride times and either much less or much more variable step width (in steady state walking) than older adults who have no falls history [9–12]. However, of these reported differences, changes in walking speed, stride length and stride width are more likely to reflect adaptations made in response to an increased fear of falling [13, 14] and a desire to increase gait stability [15], rather than representing actual mechanisms of falling. Mortaza et al. [9] suggested that differences in the variability of temporal gait parameters (e.g., stride time) were more sensitive than spatial gait parameters (e.g., stride length) when it came to distinguishing fallers from non-fallers in an older population. This notion was further supported by other research, which demonstrated that more variable stride times, walking speeds and double support times were key predictors of future falls in older adults [13, 16–18]. Collectively, these studies suggest that measures of temporal variability during gait, including stride and step timing variability, may be appropriate targets for gait training programs that seek to reduce falls risk in older adults.

Traditionally, metronomes have been used to provide rhythmic auditory cues to improve spatiotemporal gait outcomes, with previous research showing that they may improve walking stability in people with neurological conditions, such as Parkinson’s disease [19–21]. However, similar cueing methods do not appear to reduce gait variability in healthy individuals [20, 22] and may even increase stride time variability in this population [21, 23]. A possible explanation for the inconsistent results reported for healthy individuals and people with Parkinson’s disease might be that three of the studies involving older individuals were conducted solely in healthy non-fallers [20, 21, 23]. In the one study that did assess fallers, approximately 50% of the included participants had a history of falling [22]. These participants were, however, pooled with non-fallers in the analysis and, consequently, no specific conclusions could be made for the effect of cueing on gait variability in individuals with a history of falls. Research shows that gait patterns differ between fallers and non-fallers [9–11], and individuals with a history of falling are more likely to exhibit impaired movement patterns. It is, therefore, possible that auditory cueing strategies may be more effective for individuals who have a history of falling.

Although a traditional metronome is easy to use, the cue is delivered continuously and provides no information on the stability or consistency of the participant’s gait patterns. Modern biofeedback systems have the potential to address this shortcoming by providing participants with real-time feedback that automatically adapts to changes in the participant’s walking pattern. Specifically, these systems can measure an individual’s gait pattern in real-time and provide feedback based on their current movement. Participants can subsequently use this feedback to alter their gait patterns and improve the specific attributes being monitored (e.g., stride timing variability). The potential of this type of feedback was demonstrated by Begg et al. [24], who reported that adaptive visual feedback on minimum toe clearance may decrease tripping risk in older individuals. To our knowledge, the study by Begg et al. [24] is the only study to investigate the effects of incorporating adaptive feedback on the lower limb gait characteristics associated with falls risk in healthy older adults. Previous studies that have utilized adaptive feedback in older adults have mainly focused on reducing trunk sway and joint loads [25, 26], while its effects on gait variability have been largely overlooked. If cueing strategies can improve gait timing variability and assist in improving the way these individuals walk, it may be possible to reduce the risk of falls in older adults. Hence, the aim of this study was to investigate the immediate effect of two cueing strategies (traditional and adaptive metronome) on gait variability in older adults with a history of falls. Furthermore, this study sought to determine whether an adaptive metronome was superior to a traditional metronome, with respect to reducing spatiotemporal variability. We hypothesized that both cueing strategies would reduce stride time variability and that this effect would be greater for the adaptive metronome.

## Methods

This study employed a cross-over study design that adhered to the STROBE guidelines [27].

### Participants

An invitation to participate in this research was sent to all potentially eligible individuals who had previously participated in gait studies at the Australian Catholic University, Brisbane. Additionally, the study was advertised via social media and University email circulars between August and December 2018. Participant eligibility criteria were: (i) aged ≥ 65 years; (ii) able to ambulate independently; (iii) normal or corrected to normal vision (assessed at initial screening as Bailey-Lovie high contrast visual acuity ≤ 0.30 Log-MAR); and (iv) ≥ 1 fall in the past year. A fall was defined as unintentionally coming to rest on a lower surface without being exposed to an overwhelming external force or a major internal event [2]. Participants were excluded if they had: (i) significant cognitive impairment (assessed at initial screening as Standardized Mini Mental State Examination [SMMSE] total < 24); (ii) any musculoskeletal injury affecting the lower limbs or spine during the past 2 years; or (iii) a diagnosed neurological condition (e.g., Parkinson’s disease). This study was approved by the Australian Catholic University Human Research Ethics Committee (2018-130E) and all participants gave their written informed consent prior to participation.

**Fig. 1:**
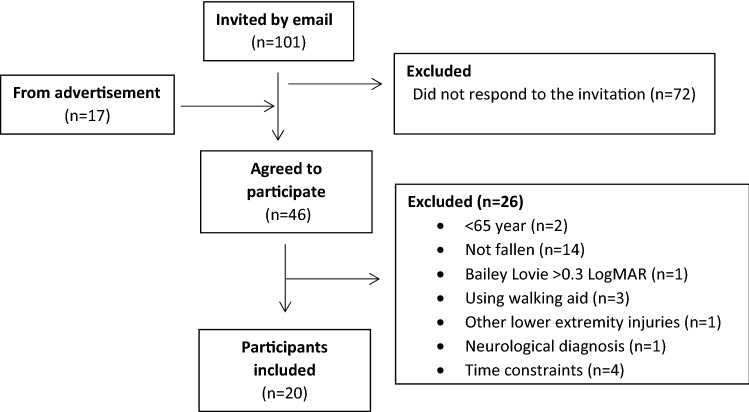
Flow chart of the inclusion process

Forty-six individuals expressed interest in the study, but 26 were excluded during the screening process, as they did not meet the inclusion/exclusion criteria (Fig. 1). The remaining 20 individuals (15 women), who had a mean age of 71 (4.9) years, were included.

### Procedure

Participants’ gait patterns were assessed in the University’s biomechanics laboratory on two occasions separated by 6–8 days. During the first session, demographic information, including age, sex and number of previous falls, was obtained via questionnaire, while height and mass were established via anthropometric measures. Additionally, participants were assessed for cognitive function, visual acuity and balance confidence, using the SMMSE, the Bailey-Lovie high contrast visual acuity test, and the 6-item Activities-specific Balance Confidence Scale (ABC-6) [28], respectively.

Participants were randomly assigned using a block randomization method, to one of the two testing protocols that determined the specific walking-based assessments they would complete during the first and second sessions. For *Protocol A,* the first testing session required participants to walk along a sealed 9-m-long walkway at a self-selected comfortable speed under 3 testing conditions: (i) 3-min walking without any form of cue (i.e., baseline gait); (ii) 6 min walking while receiving adaptive feedback regarding their step time; and (iii) 3-min walking without any form of feedback (i.e., immediate post-feedback gait). For *Protocol B*, the first testing session required participants to perform the same baseline and post-feedback gait tasks as in Protocol A, but the second condition (ii) involved the participants walking for 6 min while receiving a traditional metronome cue (set at the participant’s average step frequency determined during the baseline condition), rather than adaptive feedback. Participants who completed Protocol A during the first session, subsequently completed Protocol B for the second session, while those who completed Protocol B first, completed Protocol A during the second visit.

### Data collection

During the performance of the walking trials, participants’ movement patterns were captured and recorded using a 22-camera three-dimensional motion analysis system (Nexus 2.7, Vicon, Oxford, UK). To facilitate this, sixteen spherical reflective markers were positioned on each participant’s lower body according to the Vicon Plug-In Gait model [29, 30].

**Fig. 2:**
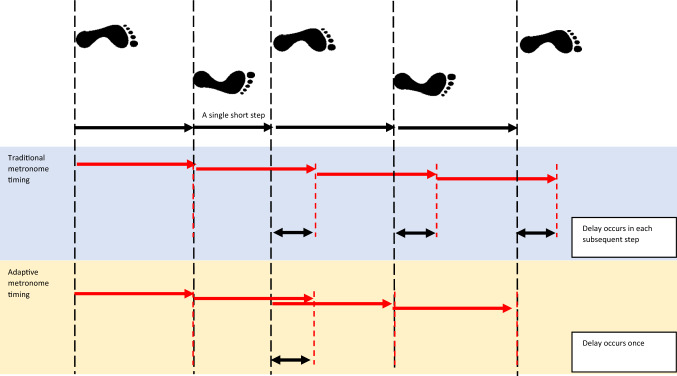
Illustration of the behavior of the two metronomes, as a consequence of one step with a shorter step time. Step times are represented with the top set of right-facing arrows (black), with heel strikes marked as vertical long dashed lines (black). The two bottom sets of right-facing arrows (red) represent the timing delays of the two metronomes, with their ‘beeps’ represented as vertical short-dashed lines (red). There is a recurring delay (double-sided arrows) that can occur with a traditional metronome as a result of a single short step, compared to an adaptive metronome that is ‘reset’ with each heel strike and, thus, the delay is mitigated for every subsequent step

Both the traditional and the adaptive metronome cues were provided to the participants via custom-written Matlab (R2018a, Mathworks Inc, Natic, USA) code at a time interval equal to the mean step time determined from their baseline walking trials. The frequency of the tone was 8 kHz and was played for a duration of 30 ms. Prior to cueing, participants were asked to ensure they could clearly hear the cue along the full distance of the walkway and volume was adjusted accordingly. The traditional metronome repeatedly played a short tone at a time interval that was equal in length to the participant’s average step time measured at baseline and, thus, sounded a tone at consistently spaced time intervals. For the adaptive metronome intervention, the metronome played an audible tone after each foot strike at a time interval that was equal to the participant’s average step time during the baseline trials. By playing the tone at a set time interval after each foot strike, the adaptive metronome intervention provided a consistently timed auditory cue for each step, regardless of whether the participant matched the feedback’s tempo (Fig. 2). Participants were instructed to walk back and forth in the lab, through a central area in which measurements took place. In providing feedback, the adaptive metronome was dependent on this measurement area and feedback was thus provided only within the central part of the walkway (6 m per walk) and not during the turns. In contrast, the traditional metronome was active constantly during the feedback phase, in both the walks and the turns. The participants were told to time their heel strikes with the tone for both metronome conditions.

To generate the timing of the audible tones for the adaptive metronome, the occurrence of foot strikes was determined in real-time by assessing the anterior–posterior velocity of the heels of the feet relative to the posterior superior iliac spine [31]. Data were collected for five trials for each of the baseline, cueing/feedback and post-cueing/feedback conditions, with one trial defined as a single passage from one end to the other end of the walkway and these data were used in all subsequent analyses.

### Data processing

Marker trajectories were identified and reconstructed in Vicon Nexus and filtered using a generalized cross-validation quintic spline with a predicted mean-squared error of 15 mm^2^ [32]. To minimize the effect of acceleration and deceleration at the start and the end of the trials, only the data from the middle five meters of each of the five trials were used in the analyses. These data were then used to derive each participant’s stride time, stride length, walking speed and duration of double leg support variability, represented by the coefficient of variation (CV; SD/Mean * 100).

### Statistical analysis

All statistical analyses were conducted using SPSS version 25 (IBM Corporation, New York, USA). All data were checked for normality by evaluating the skewness and kurtosis and visually inspecting the histograms and Q-Q plots. Pearson’s correlation coefficient and independent t-tests were used to investigate any possible associations between participant demographics (sex, age, body mass index (BMI), number of falls, SMMSE, visual acuity), fear of falling (ABC-6), and baseline gait variability. Repeated-measures ANOVA was used to assess the possible effect of each cueing strategy (2 levels) on the different gait variables between time points (3 levels; Baseline, During intervention, Post-intervention). Where a significant main effect was returned, pairwise comparisons were performed with Bonferroni adjustment. If assumptions of sphericity were violated, the Greenhouse–Geisser correction was used. A *p*-value less than 0.05 was considered statistically significant. Using stride time variability as the primary outcome measure, an a priori sample size calculation, based on a power of 80%, an alpha of 5% and a correlation among repeated measures of 0.5 indicated that at least 19 individuals were needed to determine the influence of each cueing strategy on stride time variability (effect size = 0.25).

## Results

Three of the 20 participants had received hip or knee joint replacements > 2 years prior to the sessions (Table 1). Excluding the individuals with hip and/or knee replacements did not influence the results, thus, all were included in the final analysis. All data (participant characteristics and gait-related outcomes) met the assumptions of normality and there was no significant influence of participant demographics, fear of falling, or order of cueing/feedback (i.e., traditional vs. adaptive first) on the gait variability measures (all *p*’s ≥ 0.061).

**Table 1:** Characteristics of the participants

Characteristic	*n* = 20
Women, *n* (%)	15 (75)
Age (years), mean (SD)	71 (4.9)
Body mass index (kg/m^2^), mean (SD)	28.1 (5.56)
Number of falls previous year, mean (SD)	2.2 (1.5)
Total joint replacement	
Hip, *n* (%)	1 (5.3)
Knee, *n* (%)	2 (10.5)
SMMSE score (maximum = 30), mean (SD)	29.3 (0.97)
High-contrast visual acuity (Log-MAR), mean (SD)	0.09 (0.08)
ABC-6 score (maximum = 100), mean (SD)	67.0 (25.2)
Days between sessions, mean (SD)	7 (0.32)
Baseline gait session 1	
Stride time (s)	1.07 (0.10)
Stride time variability (%)	2.13 (0.60)
Stride length (m)	1.21 (0.15)
Stride length variability (%)	2.95 (0.82)
Walking speed (m/s)	1.15 (0.20)
Walking speed variability (%)	3.27 (2.10)
Double leg support (s)	0.13 (0.03)
Double leg support variability (%)	16.17 (6.11)

*SD* standard deviation, *SMMSE* standardized mini-mental state examination, *ABC-6* 6-item activities-specific balance confidence scale

**Fig. 3:**
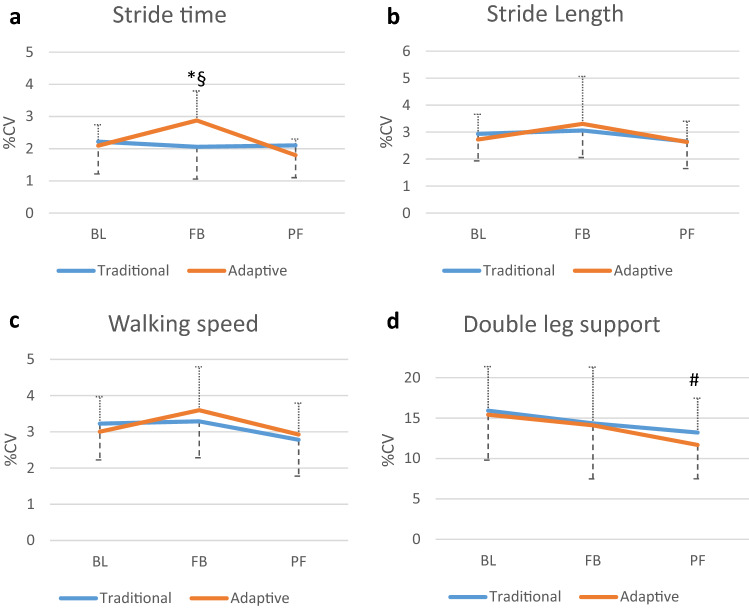
**a**–**d** Effect of time and cueing on mean gait variability. The error bars represent + or − 1 standard deviation. CV = coefficient of variation, BL = Baseline, FB = During intervention, PF = Post-intervention, Traditional = traditional metronome, Adaptive = adaptive metronome, *significant difference between Baseline and During intervention conditions, ^§^significant difference in the change between Baseline and During intervention feedback conditions between the Adaptive and Traditional metronomes, ^#^significant difference between Baseline and post intervention conditions

Mauchly’s test indicated that the assumptions of sphericity were met for all analyses (*p* > 0.05), except the cueing strategy * time point interaction for walking velocity variability (*p* = 0.043). Therefore, degrees of freedom were corrected using Greenhouse–Geisser estimates of sphericity for this interaction (*ε* = 0.77). There were significant effects of time point on stride time variability (*F* (2) = 9.83, *p* < 0.001) and duration of double leg support variability (*F* (2) 3.69, *p* = 0.034). Post-hoc tests revealed that, compared with the baseline condition, participants had significantly increased stride time variability during feedback and decreased duration of double leg support variability for the post-feedback conditions. There was also a significant time*cueing strategy effect for stride time variability (F (2) 8.08, *p* ≤ 0.001). To breakdown this interaction, post-hoc tests were performed comparing the During feedback and Post-feedback time points for the two cueing strategies against their respective baseline conditions. This indicated a significant difference in the change between Baseline and During feedback (*F* (1) = 19, *p* = 0.011), with variability increasing with the adaptive metronome relative to the traditional metronome. No other effects of time or cueing strategy on the gait variability measures were observed (Fig. 3a–d).

## Discussion

In this study, walking with the adaptive metronome led to increased stride time variability compared to both baseline and the traditional metronome, whereas both cueing conditions reduced double leg support variability post intervention. These contradicting results indicate that these two cueing strategies may have limited ability to reduce gait variability in older adults with a history of falls.

Previous research has reported auditory cues to be effective in improving different gait parameters, such as gait variability in individuals with Parkinson’s disease [19–21]. However, similar to other studies in healthy elderly non-fallers [20–23], we found only limited effects of the two cueing conditions. For this group of older adults with a history of falls, the only differences observed between the two conditions were an increase in stride time variability during the adaptive metronome walking trials and a reduction in double leg support time variability following the adaptive metronome intervention. It is well known that people living with Parkinson’s disease have trouble with the sequencing and timing of movement [33–35]. In such cases, the metronome could potentially be used to externally regulate the timing of movement. Healthy adults (fallers or otherwise) are generally not known to have problems with the timing of movement and it is possible that the cueing provided by the metronome interfered with the natural internal regulation of their movement [36]. That is, the need to time their steps to the beats in the two cueing conditions may have resulted in participants focusing too much on their performance, which in turn may have led to increased gait variability.

In the current study, stride time variability was increased during the adaptive metronome conditions, which may have also increased the participants' fall risk [13, 16–18] compared to both the baseline and traditional metronome conditions. One explanation for this may be found in a constraints-led interpretation of the coordination of walking [37]. In natural walking, performance is mainly dependent on individual constraints, such as preferred step length, and environmental constraints, such as a step or a stair that may be present on one’s planned route [38, 39]. From this viewpoint, the metronome intervention might have resulted in increased task demands; instead of freely timing one’s steps, participants were asked to time this with the beats of the metronome. Further examination of the data also revealed that the participants reduced their walking speed during the adaptive feedback condition. It is possible that the participants’ reduction in walking speed was a result of the complexity of the adaptive feedback, which in turn may have increased the variability of the gait parameters [40].

The effect of different interventions on gait variability may be dependent on the magnitude of baseline variability, which provides another possible explanation for our result; with greater baseline variability there is arguably a greater capacity to reduce this variability. Although participants with a history of falls are reported to walk with increased stride time variability compared to non-fallers, it should be noted that the participants in our study exhibited stride time variabilities at baseline that were equivalent to or lower than healthy non-fallers examined in previous research [21–23]. In a study by Roos et al. [41] that used a computer simulation model, it was concluded that increased gait variability was only related to a greater fall risk in those already exhibiting high gait variability. In contrast, there was no effect of increased gait variability on fall risk in those exhibiting low gait variability. Further studies may reveal if auditory cues using adaptive and/or traditional metronomes, influence gait variability in those exhibiting greater baseline gait variability than those included in this study.

In contrast to a previous study reporting no effect of cueing on duration of double leg support variability in healthy non-fallers [20], a reduction in this measure was observed post intervention in the current study. Since there was a trend for a reduction in double leg support variability during feedback (Fig. 3d), it may be possible that this represents a carry-over effect from the intervention that is more pronounced when the external stimulus, i.e., the cueing, was removed. However, since the two cueing strategies also introduced increased gait variability (stride time), this result may have limited clinical application.

One of the strengths of this study is that we only included older adults who had a history of falls. Individuals who have previously fallen more often exhibit impaired gait characteristics; hence, auditory cueing techniques may be more effective in this population. Moreover, individuals with a history of falls are at an elevated risk of future falls [42], and thus represent a likely target population for this intervention*.* Another strength is that we used 3D motion analysis that has high test–retest reliability [43] and is considered to be the gold standard, against which other gait assessment methods are validated [44].

This study is associated with some limitations. So as not to influence the results with different participant instructions, we used the same instruction for both the adaptive and traditional metronome protocols. However, some further instructions might have been required to understand the adaptive metronome. Since the timing of the adaptive metronome’s tones was dependent on the participant’s own walking pattern (e.g., it stopped when the participant stopped), some participants reported difficulties tuning themselves into this rhythm. Also, as we sought to target changes in step time variability, which have been associated with falls in straight-line walking, the adaptive metronome cue was only provided during the middle 6 m of the walkway where straight-line walking took place and not in the ends or during the turns. The traditional metronome cue was provided along the entire walkway including the turns, consistent with how it would be delivered in practice for gait training. It is, thus, possible that the result may have been different if alternate instructions were provided, such as “try to create a beat as consistent as possible”, or similar and/or if the traditional metronome was muted during the turns. Our results further showed that the group of older adults with a history of falls included in the current study showed lower stride time variability than healthy older adult reported elsewhere. This may be explained by the large proportion of our participants who were recruited via convenience sampling from our previous gait research. This previous study recruited participants in collaboration with a local active aging community, which may have skewed our sample to include a more active group of falling older adults, compared to the general population. Finally, while our power calculation showed that at least 19 participants were needed to examine the effect of the two metronomes on our main outcome (stride time variability), it should be acknowledged that the final sample of 20 participants may have been too small to detect any effect of the different cueing strategies on the secondary outcomes.

## Conclusion

In this study, we sought to evaluate the effect of a traditional and adaptive metronome on spatiotemporal gait variability parameters. Our results indicate that these two cueing strategies may have limited ability to reduce gait variability in older adults with a history of falls. Future studies may be warranted to evaluate the effect of different cueing strategies on gait variability measures in individuals exhibiting greater baseline variability than those included in this study.

## Data Availability

The datasets used and/or analyzed during the current study are available from the corresponding author on reasonable request.
